# TyG‐HDL Outperforms TyG Combined With Obesity Indicators in Predicting Chronic Kidney Disease Associated With High Fasting Blood Glucose Levels in the Chinese Elderly Population: A Two‐Stage Study Based on GBD 2023 and CHARLS Data

**DOI:** 10.1155/ije/8245427

**Published:** 2026-09-07

**Authors:** Letai Li, Li Long, Tingting Chen, Xinhong Tian, Can Huang, Yutong Chen, Shiying Ye, Meng Ye, Jiajie Leng, Zhenrui Cao, Yingjiu Jiang, Mengjun Bie

**Affiliations:** ^1^ Department of Cardiothoracic Surgery, The First Affiliated Hospital of Chongqing Medical University, Chongqing, China, cqmu.edu.cn; ^2^ The First Clinical College of Chongqing Medical University, Chongqing, China, cqmu.edu.cn; ^3^ Department of Nephrology, People′s Hospital of Yuechi County, Guang’an, China; ^4^ Shanghai Jiao Tong University School of Nursing, Shanghai, China; ^5^ College of Traditional Chinese Medicine, Chongqing University of Traditional Chinese Medicine, Chongqing, China; ^6^ The Second Clinical College of Chongqing Medical University, Chongqing, China; ^7^ School of Basic Medical Sciences, Chongqing Medical University, Chongqing, China, cqmu.edu.cn

**Keywords:** China Health and Retirement Longitudinal Study (CHARLS), chronic kidney disease (CKD), fasting blood glucose (FBG), metabolic dysregulation Global Burden of Disease (GBD), triglyceride-glucose (TyG) index

## Abstract

**Background:**

Chronic kidney disease (CKD) is a major public health issue in China, especially among older adults, with rising prevalence linked to metabolic disorders such as hyperglycemia. This study aimed to assess the burden of CKD attributable to elevated fasting blood glucose (FBG) in elderly Chinese, focusing on the predictive value of composite metabolic indices derived from the triglyceride‐glucose (TyG) index.

**Method:**

The study included two phases: (1) analysis of the 2023 Global Burden of Disease (GBD) database to assess the CKD burden due to hyperglycemia among adults aged ≥ 60 years in China from 1990 to 2023 and (2) evaluation using the China Health and Retirement Longitudinal Study (CHARLS) cohort, analyzing associations between 15 blood glucose‐related indices and CKD in older adults.

**Results:**

The GBD analysis revealed a significant burden of CKD due to hyperglycemia in China, with increasing mortality and disability‐adjusted life years (DALYs) projections. In the CHARLS cohort, four indices—TyG‐HDL, TCBI, FBG‐HDL, and CTI—were significantly associated with CKD. Elevated levels of these indices, particularly TyG‐HDL, demonstrated strong associations with CKD risk, with nonlinear, threshold‐dependent effects observed in restricted cubic spline analyses.

**Conclusion:**

Blood glucose‐related indices, especially TyG‐HDL, offer strong predictive value for CKD risk in older Chinese adults. These findings suggest that integrating these indices into screening programs could enhance early CKD detection and management strategies, helping mitigate the growing disease burden in aging populations.

## 1. Introduction

Chronic kidney disease (CKD) is a progressive and irreversible condition characterized by sustained reduction in glomerular filtration rate or persistent evidence of kidney damage. With a global prevalence of 10.4%, CKD constitutes a major public health challenge, disproportionately affecting low‐ and middle‐income countries and approximately 120 million adults in China [1, 2]. Over the past three decades, demographic aging and the rising incidence of comorbid chronic conditions—including diabetes mellitus and obesity—have intensified the epidemiological and clinical challenges associated with CKD prevention and management [3]. Consequently, there is an urgent need to advance risk‐stratified strategies for accurate screening, early diagnosis, and timely intervention.

Beyond its renal manifestations, CKD reflects systemic metabolic dysregulation and serves as a sentinel of multisystem pathophysiology. Importantly, the global epidemic of obesity and unhealthy dietary patterns has been increasingly recognized as a major modifiable driver of this metabolic derangement. Excessive adiposity—particularly visceral fat accumulation—promotes insulin resistance (IR), dyslipidemia, and a low‐grade chronic inflammatory state, evidenced by elevated circulating levels of proinflammatory cytokines including tumor necrosis factor‐alpha and interleukin‐6 [4]. In aging populations, this relationship is further complicated by sarcopenic obesity and age‐related fat redistribution, rendering conventional anthropometric measures such as body mass index (BMI) and waist circumference (WC), insufficient proxies for true metabolic health [5]. The interplay between adiposity‐driven IR, sympathetic overactivation, and renal hemodynamic changes directly contributes to glomerular hyperfiltration, albuminuria, and subsequent progressive kidney function decline. As such, CKD is widely recognized not only as a consequence but also as a driver of cardiovascular and metabolic morbidity [6].

In recent years, the triglyceride‐glucose (TyG) index, a validated surrogate marker of IR, has gained broad acceptance for metabolic and cardiovascular risk prediction [7]. Building upon the TyG index, 15 composite metabolic indices have been proposed to capture the combined burden of glucose, lipid, and adiposity‐related disturbances: TyG, TyG‐HDL, TCBI, FBG‐HDL, CTI, TyHGB, TyG‐BMI, TyG‐ASBI, Met‐IR, CHG, TyG‐WHtR, TyG‐WC, TyG‐WWI, TyG‐BRI, and TyG‐RFM [8, 9]. While these indices hold promise for refining metabolic risk assessment, no study to date has systematically evaluated their associations with CKD in older adults—despite CKD’s well‐established role as both a consequence and driver of metabolic derangement. Crucially, emerging evidence from large longitudinal cohorts has demonstrated that an elevated TyG index predicts a 44% to 55% higher incidence of CKD over years of follow‐up and directly correlates with accelerated progression to end‐stage kidney disease and higher all‐cause mortality in patients with established renal impairment. Furthermore, the simple triglyceride‐to‐HDL‐cholesterol ratio has been shown to increase CKD risk by 1.4 to 1.7 times, highlighting the usefulness of monitoring the comprehensive cardiometabolic profile to protect kidney function.

Despite these advances, prior studies have predominantly focused on the classical TyG index and have seldom evaluated the comparative predictive performance of its composite derivatives, especially in elderly Chinese populations where nutritional status, dietary patterns, and adiposity distribution differ markedly from Western cohorts. Moreover, the translational utility of these indices—particularly their potential to guide nutritional interventions, obesity management, and early lifestyle modification—remains underexplored. Given that dietary factors such as glycemic load, fiber intake, and fatty acid composition directly influence triglyceride, glucose, and HDL‐cholesterol levels, identifying which composite indices most robustly capture CKD risk could inform targeted, cost‐effective screening and preventive strategies in community settings.

Therefore, this study proceeds in two complementary phases. First, using the 2023 Global Burden of Disease (GBD) database, we quantified the population‐attributable burden of CKD attributable to elevated fasting blood glucose (FBG) among adults aged ≥ 60 years in China and examined its temporal trends from 1990 to 2023. Second, leveraging data from the nationally representative China Health and Retirement Longitudinal Study (CHARLS) cohort, we conducted a rigorous epidemiological analysis to evaluate the independent and joint associations of the 15 composite metabolic indices with prevalent CKD in older Chinese adults. Our primary objectives were (1) to identify robust, accessible biomarker‐based tools for CKD risk stratification that reflect obesity‐related metabolic dysregulation rather than simple anthropometry; (2) to explore threshold‐dependent, nonlinear relationships that could define actionable clinical cutoffs; and (3) to generate actionable evidence supporting integrated nutritional and lifestyle interventions, thereby informing a novel, CKD‐specific metabolic risk stratification framework that aligns with innovative prevention and management strategies in aging populations.

## 2. Method

### 2.1. Data Resources

#### 2.1.1. GBD 2023

This study utilizes publicly available data from the GBD 2023 study to assess the burden of CKD attributable to elevated FBG among individuals aged ≥ 60 years in China from 1990 to 2023. Analysis metrics include deaths, disability‐adjusted life years (DALYs), the age‐standardized mortality rate (ASMR), and the age‐standardized DALY rate (ASDR), with all data sourced from the Global Health Data Exchange platform [10]. The GBD study employs a comparative risk assessment framework [11]. By integrating high FBG exposure data from the Chinese population with relative risk evidence for CKD, it calculates population‐attributable fractions to estimate the CKD disease burden attributable to elevated FBG. The model undergoes statistical smoothing and adjustment to enhance the robustness and comparability of results [12]. This study utilizes only publicly available aggregated data and does not involve any personally identifiable information.

#### 2.1.2. CHARLS

We conducted an individual‐level analysis using the CHARLS, a nationally representative survey of Chinese adults aged 60 years and older that collects information on sociodemographic characteristics, health conditions, lifestyle factors, physical measurements, and selected laboratory tests. The 2015 wave served as the analytic baseline for this cross‐sectional study, while the 2011 wave was used to support participant linkage and data consistency checks [13].

Participants were eligible if they were successfully linked across the 2011 and 2015 waves; had available information to define CKD; had anthropometric measurements (height, weight, and WC) and laboratory measures required to compute at least one of the 15 indicators composed of glucose levels; and had complete data on key covariates. We excluded individuals with missing CKD information, missing key anthropometric measurements, or missing key covariates. After applying these criteria, 4050 participants were included in the final analytic sample.

### 2.2. Statistical Analysis

#### 2.2.1. GBD Analysis

##### 2.2.1.1. Descriptive and Trend Analysis

This study conducted a descriptive analysis and quantitative assessment of the changing trends in the disease burden of CKD attributable to elevated FBG among individuals aged ≥ 60 years in China from 1990 to 2023 [14]. Key indicators analyzed include deaths, DALYs, and their corresponding age‐standardized rates (ASMR and ASDR). To quantify the pace and direction of long‐term trends, average annual percentage changes (AAPC) and their 95% confidence intervals (CIs) were calculated. The AAPC, derived from the joinpoint regression model, represents the geometric weighted average of the annual percentage change (APC) across the identified trend segments over the entire study period [15]. The criteria for determining trend direction are as follows: if both the AAPC and the upper limit of its 95% CI are less than 0, the trend is downward. If the lower limit of the 95% CI is greater than 0, the trend is upward. If the 95% CI includes 0, the trend is considered stable. All calculations were performed using R software.

##### 2.2.1.2. Joinpoint Regression and AAPC

To identify key turning points in the long‐term trends of disease burden from 1990 to 2023, this study employed joinpoint regression analysis [16]. This model segments time series into consecutive segments and identifies statistically significant “turning points” where trends undergo marked shifts, thereby providing a more granular description of the phased characteristics of burden evolution. Within each trend phase identified by the model, the annual percentage change and its 95% CI were calculated to characterize the local rate of change during that phase. Concurrently, the average annual percentage change and its 95% CI were computed for the entire study period (1990–2023) to reflect the magnitude and direction of the overall burden trend [17]. The criteria for determining trend direction were consistent with those for the AAPC. All analyses were performed using the Joinpoint Regression Program software.

##### 2.2.1.3. Decomposition Analysis

To identify the drivers of disease burden changes between 1990 and 2023, this study employed a demographic decomposition method [18]. This approach breaks down the overall change in deaths and DALYs into contributions from three independent drivers: population growth, aging age structure, and changes in age‐specific disease risks. By quantifying each factor’s independent contribution to burden growth, the study aims to distinguish the respective roles of demographic dynamics and genuine epidemiological risk shifts, thereby revealing the primary drivers behind absolute burden increases. This methodology aids in understanding the structural causes of burden changes and provides evidence for targeted interventions.

##### 2.2.1.4. BAPC Prediction

To project trends in the burden of CKD attributable to elevated FBG among individuals aged ≥ 60 years in China from 2024 to 2040, this study employed a Bayesian age‐period‐cohort model for extrapolation [19]. The model, grounded in historical data from 1990 to 2023, employs a random walk prior to constrain the smoothness across age, period, and birth cohort dimensions, assuming that recently observed period and cohort effects will persist. Model parameters were fitted using Markov chain Monte Carlo methods, with convergence assessed via Gelman–Rubin statistics [20]. Final projections will provide median estimates for ASMRs, DALY rates, and their 95% uncertainty intervals between 2024 and 2040. Absolute burden projections for deaths and DALYs will also be generated to assess the direction and magnitude of future disease burden shifts.

#### 2.2.2. CHARLS Analysis

##### 2.2.2.1. Definition

The primary endpoint of this study was defined as CKD occurring during the observation period from 2011 to 2015. CKD was identified based on [21] an estimated glomerular filtration rate (eGFR) of < 60 mL/min/1.73 m^2^.

The exposures of interest were 15 Glu‐related indices combining the triglyceride–glucose (TyG) index with anthropometric or laboratory measures. TG, FBG, HDL‐C, and CRP were measured from fasting blood samples in CHARLS; height, weight, and WC were measured using standardized protocols. TyG and related indices were computed as follows (units shown in each formula):

 TyG: TyG = ln[(TG (mg/dL) × FBG (mg/dL))/2] [16].
 CTI: CTI = 0.412 × ln[CRP (mg/L)] + ln[(TG (mg/dL) × FBG (mg/dL))/2] [17].
 ABSI: ABSI = WC (m)/(BMI^(2/3)^ × height (m)^(1/2)^) [18].
 TyG‐ABSI: TyG‐ABSI = TyG × ABSI [18, 19].
 BMI: BMI = weight (kg)/height (m)^2^ [20].
 TyG‐BMI: TyG‐BMI = TyG × BMI [19].
 BRI: BRI = 364.2 − 365.5 × √{1 − [(WC/(2π))^2^/(0.5 × height)^2^]}. (WC and height in meters) [22].
 TyG‐BRI: TyG‐BRI = TyG × BRI [19, 22].
 Relative fat mass (RFM): RFM (%) = 64 − 20 × (height/WC) + 12 × sex, where sex = 0 for men and 1 for women (height and WC measured in the same units, e.g., cm) [23].
 TyG‐RFM: TyG‐RFM = TyG × RFM. We followed previous CHARLS research applying TyG‐RFM as an obesity‐related TyG indicator [23, 24].
 TyG‐HDL: TyG‐HDL = TyG/HDL‐C (mg/dL) [25, 26].
 TCBI: TCBI = TG (mg/dL) × total cholesterol (mg/dL) × body weight (kg)/1000 [27, 28].
 TyG‐WC: TyG‐WC = TyG × WC (cm) [19].
 WHtR: WHtR = WC (cm)/height (cm) [22].
 TyG‐WHtR: TyG‐WHtR = TyG × WHtR [19].
 WWI: WWI = WC (cm)/√weight (kg) [28].
 TyG‐WWI: TyG‐WWI = TyG × WWI [19, 28].
 TyHGB: TyHGB = TG (mmol/L)/HDL‐C (mmol/L)] + 0.7 × FBG (mmol/L) + 0.1 × BMI (kg/m^2^) [29].
 FBG/HDL: FBG/HDL = FBG (mg/dL) divided by HDL‐C (mg/dL) [30].
 Mets‐IR: metabolic score for IR (METS‐IR) = ln [(2  ×  fasting glucose + TG)  ×  BMI]/[ln (HDL‐C)] [31].
 CHG: CHG = Ln [(TC × FBG)/(2 × HDL)] [32].

##### 2.2.2.2. Covariates

Covariates included age, sex, education, residential location (urban/rural), marital status, smoking status, alcohol drinking, and sleep duration. We additionally adjusted for SBP and DBP to account for blood pressure variability when modeling associations between the indices and CKD.

##### 2.2.2.3. Statistical Analysis

Associations between each Glu‐related index and CKD were examined using logistic regression. For comparability across indices, we reported ORs and 95% CIs per IQR increase. Three models were fitted: Model 1 (M1), unadjusted; Model 2 (M2), adjusted for age, sex, education level, residential location, and marital status; and Model 3 (M3), further adjusted for smoking status, alcohol drinking status, sleep duration, systolic blood pressure (SBP), and diastolic blood pressure (DBP). The fully adjusted model (M3) was considered the primary model.

Potential nonlinear dose–response relationships were evaluated using restricted cubic splines (RCSs). Discriminatory ability of each index for identifying prevalent CKD was assessed using ROC curves and AUC. Prespecified subgroup analyses were conducted by age (< 65/≥ 65 years), sex, residence, education, marital status, smoking, alcohol drinking, and sleep duration; interaction terms were used to test effect modification. Two‐sided *p* values < 0.05 were considered statistically significant.

## 3. Result

### 3.1. GBD Result

#### 3.1.1. Current Burden of Hyperglycaemia‐Related CKD in China, 2023

According to GBD 2023 data, CKD attributable to hyperglycaemia resulted in 46,981.38 deaths in China in 2023 (95% UI: 26,378.95–72,668.08). With an ASMR of 16.87 per 100,000 population (95% UI: 9.42–26.13); DALYs amounted to 1,277,922.69 (95% UI: 772,195.69–1,872,568.70), with an ASDR of 441.55 per 100,000 population (95% UI: 265.93–647.40) (Table 1). Gender disparities existed in disease burden, with higher rates observed among males than females (ASMR: 17.94 vs. 16.02 per 100,000; ASDR: 471.95 vs. 415.97 per 100,000). Between 1990 and 2023, the age‐standardized burden across the entire population showed a declining trend, with AAPC of −1.33% for ASMR and −1.04% for ASDR. The decline was greater among males than females (ASMR AAPC: −1.44% vs. −1.19%). Results indicate that despite the overall decline in age‐standardized burden of hyperglycaemia‐related CKD in China, men continue to face higher disease risk, while improvements in the burden among women remain relatively slow, warranting attention in prevention and control strategies (Supporting Table 1).

**TABLE 1:** Disease burden of hyperglycaemia‐related conditions in China: Deaths and DALYs, 1990 and 2023.

	**1990**		**2023**		**AAPC**
	**Number**	**ASMR**	**Number**	**ASMR**	
Both	22,514.53 (10,480.58–39427.76)	26.9 (12.47–46.85)	46,981.38 (26,378.95–72668.08)	16.87 (9.42–26.13)	−1.33 (−1.43 to −1.24)
Male	11,392.28 (4944.53–22123.55)	31.64 (13.73–60.82)	22,513.48 (11,618.09–36077.91)	17.94 (9.25–28.95)	−1.44 (−1.64 to −1.27)
Female	11,122.26 (4946.51–19924.27)	23.97 (10.69–42.81)	24,467.9 (12,684.19–40298.17)	16.02 (8.28–26.34)	−1.19 (−1.29 to −1.09)

	**Number**	**ASDR**	**Number**	**ASDR**	**AAPC**

Both	601,360.34 (312,692.3–1027242.17)	639.7 (331.37–1087.08)	1,277,922.69 (772,195.69–1872,568.7)	441.55 (265.93–647.4)	−1.04 (−1.11 to −0.98)
Male	305,068.11 (151,558.01–543630.08)	715.99 (353.46–1265.42)	635,645.64 (369,470.68–934255.11)	471.95 (274.26–695.41)	−1.16 (−1.25 to −1.09)
Female	296,292.23 (151,925.19–494564.07)	587.77 (300.81–981.03)	642,277.05 (369,189.92–985020.39)	415.97 (238.83–637.13)	−0.99 (−1.05 to −0.93)

#### 3.1.2. Analysis of Long‐Term Trends and Inflection Points in the Burden of Hyperglycaemia‐Related CKD in China, 1990–2023

**FIGURE 1:**
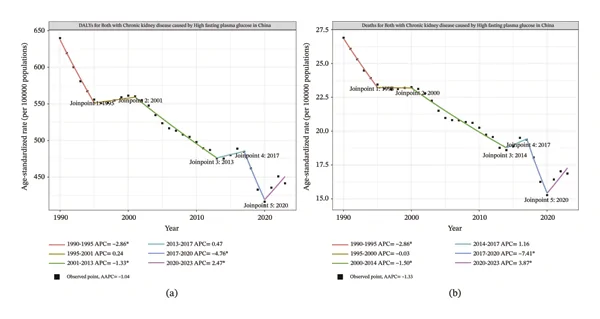
Joinpoint regression analysis of the burden of chronic kidney disease attributable to hyperglycaemia in China, 1990–2023. This figure illustrates the long‐term trend and key turning points in the ASMR for chronic kidney disease attributable to hyperglycaemia in China from 1990 to 2023. Joinpoint analysis indicates a sustained and significant downward trend in ASMR throughout the observation period, declining from 650 per 100,000 population in 1990 to 200 per 100,000 in 2020, reflecting effective disease burden control during this era. ^∗^Denotes statistically significant slope changes in trends (*p* < 0.05). ASMR: age‐standardized mortality rate; APC: annual percentage change.

This study employed a nodal regression model to analyze that the age‐standardized burden of hyperglycaemia‐related CKD in China exhibited an overall downward trend between 1990 and 2023, with a greater decline observed among males than females (AAPC mortality rate: males −1.44%, females −1.19%; DALY rate AAPC: males −1.16%, females −0.99%). The ASMR underwent multiple fluctuating phases, with the most pronounced decline occurring between 2017 and 2020 (APC = −7.41%) and the most significant increase occurring between 2020 and 2023 (APC = 3.87%). The ASDR exhibited a similar pattern, with the greatest decline occurring between 2017 and 2020 (APC = −4.76%) and the largest increase occurring between 2020 and 2023 (APC = 2.47%). The findings indicate that while the overall long‐term burden has improved, recent fluctuations suggest a leveling off or even a potential rebound in trends. The faster decline in the male burden suggests differences in risk factors or disease management between the sexes (Figure 1, Supporting Figures 1 and 2).

#### 3.1.3. Decomposition Analysis of Factors Driving Changes in the Burden of Hyperglycaemia‐Related CKD in China From 1990 to 2023

**FIGURE 2:**
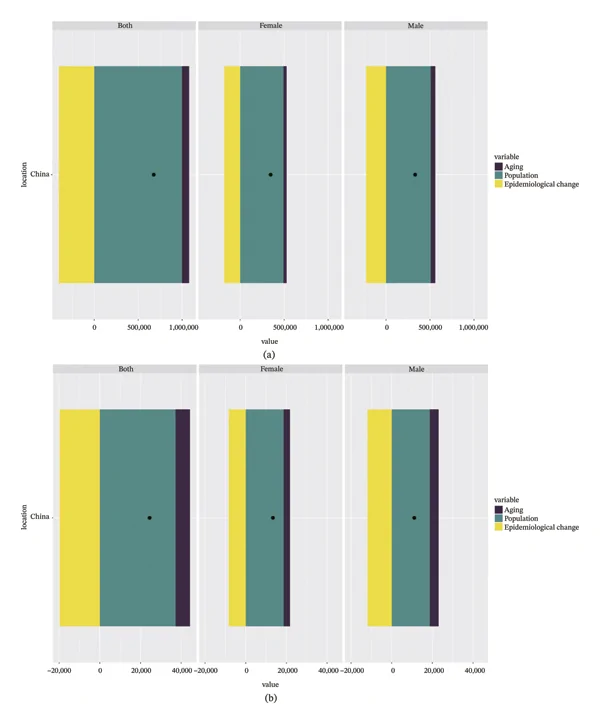
Decomposition analysis of factors driving changes in the burden of chronic kidney disease associated with hyperglycaemia in China, 1990–2023. This figure illustrates the contributions of population aging, population growth, and epidemiological shifts to variations in disease burden. Results indicate that demographic factors constitute the primary driver of the rising burden, while epidemiological improvements—such as advances in diagnosis and treatment—have exerted an effective counterbalancing effect. This suggests that, against the backdrop of ongoing demographic shifts, enhanced prevention and control measures remain essential to mitigate the disease burden.

To identify the drivers of changes in disease burden, this study employed a demographic decomposition analysis (Figure 2). Results indicate that between 1990 and 2023, the number of deaths attributable to CKD due to hyperglycaemia increased by 24,466.85 cases. This change was primarily driven by population growth (contribution rate: 152.26%), followed by aging (contribution rate: 100.00%). From 1990 to 2023, the number of CKD deaths attributable to hyperglycaemia nationwide increased by 24,466.85 cases. This change was primarily driven by population growth (contribution rate: 152.26%), with aging contributing 28.62%. Epidemiological changes (reflecting age‐specific risk levels) contributed 80.88%, indicating an overall improvement in age‐specific mortality risk. The increase in DALYs amounted to 676,562.35 person‐years, with population growth contributing 147.50%, aging contributing 12.02%, and epidemiological changes contributing 59.53%. Gender‐stratified analysis revealed significant heterogeneity (Supporting Table 2): population growth contributed most to increased mortality among males (168.58%) compared to females (139.60%); epidemiological changes contributed 107.08% and 62.83% to male and female mortality increases, respectively, indicating reduced age‐specific risks, with more pronounced improvements among males. These findings suggest that the rise in absolute burden of hyperglycaemia‐related CKD over the past three decades in China primarily stems from population expansion, while widespread improvements in age‐specific disease risks (particularly among males) partially offset the burden pressure from population growth.

#### 3.1.4. Bayesian Age‐Period Cohort Model Prediction Analysis of the Burden of Hyperglycaemia‐Related CKD in China From 2024 to 2040

**FIGURE 3:**
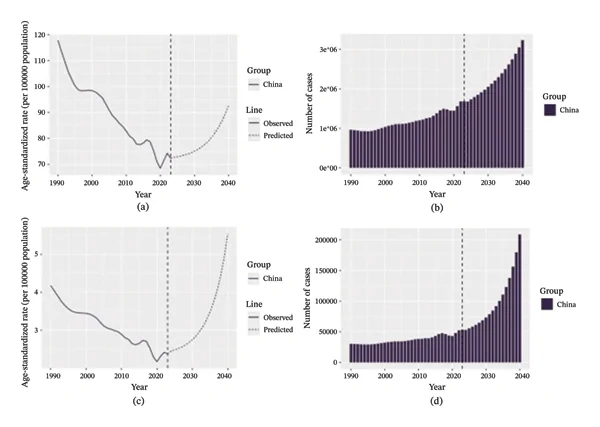
BAPC model projections of the burden of hyperglycaemia‐related chronic kidney disease in China, 2024–2040. This figure, based on a BAPC model, illustrates historical trends and future projections for the number of cases and age‐standardized rates of hyperglycaemia‐related chronic kidney disease in China from 1990 to 2040. The model indicates a sustained downward trajectory in disease burden, with projections suggesting further reduction by 2040. The BAPC model dissects the contributions of age, period, and cohort effects to long‐term trends, providing quantitative evidence for formulating forward‐looking prevention and control strategies.

Based on data from 1990 to 2023, this study employed a Bayesian age‐period cohort model to predict the burden of hyperglycaemia‐related CKD in China from 2024 to 2040 (Figure 3). Results indicate that total deaths are projected to rise from approximately 53,000 in 2024 to approximately 209,000 in 2040, while total DALYs will increase from approximately 1.68 million to approximately 32.36 million (Supporting Table 3). ASMRs remained relatively stable (2.45 per 100,000 in 2024, 5.55 per 100,000 in 2040), whereas DALY rates continued to rise (72.72 per 100,000 in 2024, 92.79 per 100,000 in 2040) (Supporting Table 4). Gender projections reveal marked disparities: by 2040, male deaths are projected to reach 72,000 (ASMR 1.86 per 100,000), while female deaths are projected to reach 136,700 (3.60 per 100,000); DALY rates for both sexes remain elevated (67.96 per 100,000 for males, 77.71 per 100,000 for females). Findings indicate that the absolute burden of hyperglycaemia‐related CKD in China will substantially increase in the future (Supporting Figures 3 and 4). Although ASMRs are projected to stabilize, the intensity of health loss attributable to the disease will continue to rise. The absolute burden growth pressure is more pronounced for females, suggesting that prevention and control efforts should prioritize population growth and gender disparities.

### 3.2. CHARLS Results

#### 3.2.1. Baseline Characteristics

A total of 4050 participants were included in this study, among whom 822 (20.3%) were diagnosed with CKD (Table 2). Compared with participants without CKD, those with CKD exhibited statistically significant differences across multiple demographic and lifestyle characteristics, as well as key clinical and metabolic parameters. Demographically, CKD patients were significantly older (68.2 ± 6.4 vs. 66.5 ± 5.5 years; *p* < 0.001) and more likely to be males (55.1% vs. 49.7%; *p* = 0.006). No significant differences were observed in education level (*p* = 0.907) or marital status (*p* = 0.260). However, urban residency was markedly more prevalent among CKD patients (24.0% vs. 18.2%; *p* < 0.001). Regarding lifestyle factors, current smoking prevalence did not differ significantly between groups (31.2% vs. 32.0%; *p* = 0.031), but the proportion of former smokers was significantly higher in the CKD group (13.2% vs. 10.0%; *p* < 0.001), suggesting potential post‐diagnosis smoking cessation behavior. Alcohol consumption showed no significant intergroup difference (*p* = 0.161), with approximately 72.8% of all participants reporting never having consumed alcohol. Mean sleep duration was significantly shorter in the CKD group (5.9 ± 2.1 h vs. 6.2 ± 2.0 h; *p* < 0.001). Clinically, systolic blood pressure was elevated in CKD patients (135.9 ± 23.5 mmHg vs. 133.0 ± 21.7 mmHg; *p* = 0.002), whereas diastolic blood pressure did not differ significantly (*p* = 0.115). Among 15 metabolic parameters assessed, four TyG‐derived indices demonstrated robust associations with CKD: TyG‐HDL, TCBI, FBG‐HDL, and CTI. Specifically, mean TyG‐HDL values were identical numerically (0.2 ± 0.1 in both groups) yet differed significantly (*p* < 0.001), reflecting greater dispersion or skewness in the CKD group; accordingly, the proportion of participants in the highest quartile (Q4) of TyG‐HDL was significantly elevated in the CKD group (27.9% vs. 21.0%; *p* < 0.005). TCBI was substantially higher in CKD patients (1713.5 ± 2262.8 vs. 1452.5 ± 1292.4; *p* < 0.001), with a correspondingly higher Q4 prevalence (26.6% vs. 20.8%; *p* = 0.026). FBG‐HDL was also significantly elevated (5.5 ± 3.7 vs. 5.0 ± 2.4; *p* < 0.001), and its Q4 proportion was markedly increased in the CKD group (30.1% vs. 23.1%; *p* < 0.001). Finally, CTI was modestly but significantly higher in CKD patients (8.9 ± 0.9 vs. 8.8 ± 0.8; *p* < 0.001), and the combined proportion of participants in the upper two quartiles (Q3 + Q4) was significantly greater (56.8% vs. 51.9%; *p* = 0.020). Collectively, these findings indicate that elevated levels of multiple TyG‐derived metabolic indices are consistently associated with CKD, supporting their potential utility as integrative markers of metabolic dysregulation in this population.

**TABLE 2:** Baseline characteristics of patients with CKD.

	Level	Overall	0	1	*p*
*n*		4050	3228	822	

CKD (mean (SD))		0.2 (0.4)	0.0 (0.0)	1.0 (0.0)	< 0.001

Age (mean (SD))		66.8 (5.8)	66.5 (5.5)	68.2 (6.4)	< 0.001

Sex (%)	Female	1994 (49.2)	1625 (50.3)	369 (44.9)	0.006
	Male	2056 (50.8)	1603 (49.7)	453 (55.1)	

Education (%)	College or above	152 (3.8)	119 (3.7)	33 (4.0)	0.907
	High school	557 (13.8)	444 (13.8)	113 (13.7)	
	Primary school or below	3341 (82.5)	2665 (82.6)	676 (82.2)	

Location (%)	City/town	786 (19.4)	589 (18.2)	197 (24.0)	< 0.001
	Village	3264 (80.6)	2639 (81.8)	625 (76.0)	

Marital (%)	Married	3290 (81.2)	2634 (81.6)	656 (79.8)	0.260
	Never‐married/separated/widowed	760 (18.8)	594 (18.4)	166 (20.2)	

Smoking (%)	Current smoker	1265 (31.9)	1016 (32.0)	249 (31.2)	0.031
	Ex‐smoker	421 (10.6)	316 (10.0)	105 (13.2)	
	Nonsmoker	2283 (57.5)	1840 (58.0)	443 (55.6)	

Drinking (%)	Drinking but less than once a month	262 (7.0)	201 (6.7)	61 (8.0)	0.161
	Drinking more than once a month	764 (20.3)	625 (20.8)	139 (18.2)	
	Never	2743 (72.8)	2179 (72.5)	564 (73.8)	

Sleep_time (mean (SD))		6.2 (2.0)	6.2 (2.0)	5.9 (2.1)	< 0.001

SBP (mean (SD))		133.6 (22.1)	133.0 (21.7)	135.9 (23.5)	0.002

DBP (mean (SD))		74.5 (11.8)	74.4 (11.6)	75.2 (12.6)	0.115

TyG‐HDL (mean (SD))		0.2 (0.1)	0.2 (0.1)	0.2 (0.1)	< 0.001

TyG‐HDL_Quantiles (%)	Q1	787 (27.6)	643 (28.2)	144 (25.1)	< 0.005
	Q2	734 (25.7)	600 (26.3)	134 (23.3)	
	Q3	696 (24.4)	560 (24.6)	136 (23.7)	
	Q4	638 (22.3)	478 (21.0)	160 (27.9)	

TCBI (mean (SD))		1505.5 (1542.8)	1452.5 (1292.4)	1713.5 (2262.8)	< 0.001

TCBI_Quantiles (%)	Q1	789 (27.7)	637 (28.1)	152 (26.3)	0.026
	Q2	728 (25.6)	588 (25.9)	140 (24.2)	
	Q3	704 (24.7)	572 (25.2)	132 (22.8)	
	Q4	626 (22.0)	472 (20.8)	154 (26.6)	

FBG‐HDL (mean (SD))		5.1 (2.7)	5.0 (2.4)	5.5 (3.7)	< 0.001

FBG‐HDL_Quantiles (%)	Q1	744 (26.1)	603 (26.4)	141 (24.6)	< 0.001
	Q2	738 (25.8)	613 (26.9)	125 (21.8)	
	Q3	672 (23.5)	537 (23.5)	135 (23.5)	
	Q4	701 (24.6)	528 (23.1)	173 (30.1)	

CTI (mean (SD))		8.8 (0.8)	8.8 (0.8)	8.9 (0.9)	< 0.001

CTI_Quantiles (%)	Q1	710 (22.5)	587 (23.3)	123 (19.4)	0.020
	Q2	827 (26.2)	676 (26.8)	151 (23.9)	
	Q3	836 (26.5)	654 (25.9)	182 (28.8)	
	Q4	781 (24.8)	604 (24.0)	177 (28.0)	

TyHGB (mean (SD))		83.0 (24.9)	82.5 (23.8)	84.9 (29.0)	0.050

TyHGB_Quantiles (%)	Q1	586 (22.9)	468 (22.9)	118 (22.6)	0.341
	Q2	642 (25.1)	525 (25.7)	117 (22.5)	
	Q3	631 (24.6)	501 (24.6)	130 (25.0)	
	Q4	702 (27.4)	546 (26.8)	156 (29.9)	

TyG‐BMI (mean (SD))		205.5 (144.8)	203.9 (132.9)	211.9 (184.3)	0.234

TyG‐BMI_Quantiles (%)	Q1	853 (30.1)	691 (30.6)	162 (28.2)	0.102
	Q2	729 (25.7)	593 (26.3)	136 (23.7)	
	Q3	653 (23.0)	517 (22.9)	136 (23.7)	
	Q4	598 (21.1)	457 (20.2)	141 (24.5)	

TyG (mean (SD))		8.7 (0.6)	8.7 (0.6)	8.7 (0.7)	0.020

TyG_Quantiles (%)	Q1	799 (25.3)	645 (25.6)	154 (24.3)	0.201
	Q2	816 (25.9)	664 (26.3)	152 (24.0)	
	Q3	809 (25.6)	648 (25.7)	161 (25.4)	
	Q4	730 (23.1)	564 (22.4)	166 (26.2)	

TyG‐ASBI (mean (SD))		0.7 (0.1)	0.7 (0.1)	0.7 (0.1)	0.785

TyG‐ASBI_Quantiles (%)	Q1	478 (18.7)	381 (18.7)	97 (18.7)	0.626
	Q2	620 (24.3)	504 (24.7)	116 (22.4)	
	Q3	684 (26.8)	545 (26.8)	139 (26.8)	
	Q4	774 (30.3)	607 (29.8)	167 (32.2)	

Met‐IR (mean (SD))		35.6 (25.3)	35.1 (23.3)	37.3 (32.1)	0.082

Met‐IR_Quantiles (%)	Q1	778 (30.4)	630 (30.9)	148 (28.4)	0.003
	Q2	646 (25.2)	536 (26.3)	110 (21.1)	
	Q3	578 (22.6)	457 (22.4)	121 (23.2)	
	Q4	559 (21.8)	417 (20.4)	142 (27.3)	

CHG (mean (SD))		5.3 (0.4)	5.3 (0.4)	5.4 (0.5)	0.002

CHG_Quantiles (%)	Q1	715 (25.0)	585 (25.6)	130 (22.6)	< 0.001
	Q2	711 (24.9)	568 (24.9)	143 (24.9)	
	Q3	703 (24.6)	583 (25.6)	120 (20.9)	
	Q4	726 (25.4)	545 (23.9)	181 (31.5)	

TyG‐WhTR (mean (SD))		4.7 (1.0)	4.7 (1.0)	4.8 (1.2)	0.392

TyG‐WhTR_Quantiles (%)	Q1	694 (24.5)	550 (24.3)	144 (25.0)	0.131
	Q2	688 (24.3)	557 (24.6)	131 (22.7)	
	Q3	694 (24.5)	567 (25.1)	127 (22.0)	
	Q4	760 (26.8)	586 (25.9)	174 (30.2)	

TyG‐WC (mean (SD))		737.6 (132.1)	735.7 (127.6)	745.0 (148.2)	0.13

TyG‐WC_Quantiles (%)	Q1	730 (25.6)	584 (25.7)	146 (25.3)	0.056
	Q2	706 (24.8)	582 (25.6)	124 (21.5)	
	Q3	697 (24.5)	557 (24.5)	140 (24.3)	
	Q4	714 (25.1)	547 (24.1)	167 (28.9)	

TyG‐WWI (mean (SD))		98.1 (13.6)	98.2 (12.9)	98.0 (16.0)	0.835

TyG‐WWI_Quantiles (%)	Q1	520 (20.3)	406 (19.9)	114 (22.0)	0.368
	Q2	624 (24.4)	509 (24.9)	115 (22.2)	
	Q3	647 (25.3)	522 (25.6)	125 (24.1)	
	Q4	771 (30.1)	606 (29.7)	165 (31.8)	

TyG‐BRI (mean (SD))		39.2 (47.9)	38.8 (43.4)	40.8 (62.5)	0.413

TyG‐BRI_Quantiles (%)	Q1	632 (24.7)	498 (24.4)	134 (25.7)	0.178
	Q2	601 (23.5)	485 (23.8)	116 (22.3)	
	Q3	623 (24.3)	511 (25.0)	112 (21.5)	
	Q4	706 (27.6)	547 (26.8)	159 (30.5)	

TyG‐RFM (mean (SD))		278.5 (112.4)	280.8 (104.8)	269.8 (138.0)	0.046

TyG‐RFM_Quantiles (%)	Q1	720 (28.1)	565 (27.7)	155 (29.8)	0.634
	Q2	646 (25.2)	515 (25.2)	131 (25.1)	
	Q3	534 (20.8)	435 (21.3)	99 (19.0)	
	Q4	662 (25.8)	526 (25.8)	136 (26.1)	

BMI (mean (SD))		23.9 (31.4)	23.9 (34.0)	23.9 (17.9)	0.966

WC (mean (SD))		84.6 (12.2)	84.6 (11.8)	84.6 (13.8)	0.910

#### 3.2.2. Associations of TyG‐Related Indices With Circadian Syndrome

According to the three‐model analysis table (Table 3) (Supporting Table 5), there were significant differences in the strength and stability of the association between 15 TyG‐related metabolic indicators and the risk of CKD. In the fully adjusted model (Model 3), CTI (per IQR: OR = 1.18, 95%CI: 1.03–1.35, *p* = 0.015), TCBI (OR = 1.11, 95% CI: 1.03–1.19, *p* = 0.004), TYG‐HDL (OR = 1.19, 95% CI: 1.06–1.33, *p* = 0.002), and FBG‐HDL (OR = 1.12, 95% CI: 1.04–1.20, *p* = 0.002) were still significantly positively associated with the risk of CKD in a dose‐response relationship (*p* for trend< 0.05). Among them, TYG‐HDL had the strongest effect, and the OR value of Q4 was 1.31 (95% CI: 0.98–1.76, *p* = 0.07) compared with Q1. Met‐IR was significant in Q4 of Model 3 (OR = 1.41, *p* = 0.025), but the trend test was only marginally significant (*p* = 0.012). TyG‐BMI and TyG were significant in Model 1 and Model 2, but the effects were weakened after full adjustment (*p* > 0.05). The remaining indicators (TyG‐WWI, TyG‐WHtR, TyG‐ASBI, TyG‐WC, TyG‐BRI, TyHGB, CHG, TyG‐RFM) were not significantly associated in any of the models. The above results indicate that CTI and TCBI integrating blood lipid and obesity indicators, as well as TyG‐HDL and FBG‐HDL integrating blood glucose and blood lipid, have more robust predictive value for CKD, while the indicators based on TyG alone or derived indicators combined with body fat percentage and WC have relatively limited predictive efficacy.

**TABLE 3:** TyG‐related metabolic indices and CKD risk: Three‐model logistic regression.

	Model 1	*p*	Model 2	*p*	Model 3	*p*
CTI per IQR	1.23 (1.10, 1.38)	< 0.001	1.25 (1.11, 1.40)	< 0.001	1.18 (1.03, 1.35)	0.015
Quartiles of CTI						
Q1	Ref	NA	Ref	NA	Ref	NA
Q2	1.07 (0.82, 1.39)	0.633	1.04 (0.80, 1.35)	0.789	0.89 (0.66, 1.19)	0.414
Q3	1.33 (1.03, 1.72)	0.029	1.35 (1.04, 1.75)	0.023	1.16 (0.87, 1.54)	0.318
Q4	1.40 (1.08, 1.81)	0.010	1.39 (1.07, 1.81)	0.013	1.14 (0.85, 1.53)	0.375
*p* of trend	0.003		0.003		0.139	
TCBI per IQR	1.11 (1.05, 1.19)	< 0.001	1.13 (1.06, 1.21)	< 0.001	1.11 (1.03, 1.19)	0.004
Quartiles of TCBI						
Q1	Ref	NA	Ref	NA	Ref	NA
Q2	1.00 (0.77, 1.29)	0.987	1.05 (0.81, 1.35)	0.737	0.90 (0.68, 1.18)	0.436
Q3	0.97 (0.75, 1.25)	0.800	1.01 (0.78, 1.32)	0.936	0.94 (0.71, 1.24)	0.659
Q4	1.37 (1.06, 1.76)	0.016	1.46 (1.12, 1.90)	0.005	1.35 (1.02, 1.79)	0.038
*p* of trend	0.033		0.012		0.005	
TyG‐HDL per IQR	1.26 (1.14, 1.39)	< 0.001	1.26 (1.14, 1.39)	< 0.001	1.19 (1.06, 1.33)	0.002
Quartiles of TyG‐HDL						
Q1	Ref	NA	Ref	NA	Ref	NA
Q2	1.00 (0.77, 1.29)	0.983	1.00 (0.77, 1.30)	0.999	0.97 (0.72, 1.29)	0.819
Q3	1.08 (0.84, 1.41)	0.542	1.09 (0.84, 1.42)	0.506	0.97 (0.73, 1.31)	0.865
Q4	1.49 (1.16, 1.93)	0.002	1.47 (1.14, 1.91)	0.003	1.31 (0.98, 1.76)	0.070
*p* of trend	0.002		0.003		0.049	
FBG‐HDL per IQR	1.15 (1.07, 1.22)	< 0.001	1.15 (1.08, 1.23)	< 0.001	1.12 (1.04, 1.20)	0.002
Quartiles of FBG‐HDL						
Q1	Ref	NA	Ref	NA	Ref	NA
Q2	0.87 (0.67, 1.14)	0.313	0.88 (0.67, 1.15)	0.354	0.91 (0.68, 1.23)	0.552
Q3	1.08 (0.83, 1.40)	0.589	1.11 (0.85, 1.44)	0.459	1.10 (0.81, 1.48)	0.535
Q4	1.40 (1.09, 1.80)	0.008	1.37 (1.06, 1.77)	0.015	1.22 (0.91, 1.64)	0.177
*p* of trend	0.003		0.004		0.042	

#### 3.2.3. RCS Analyses

**FIGURE 4:**
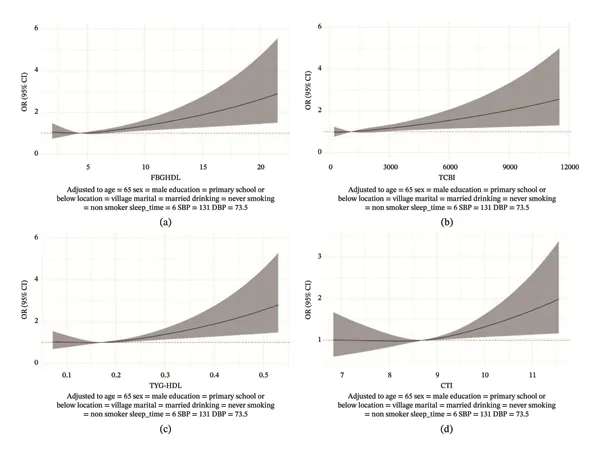
RCS.

RCS analyses (Figure 4) demonstrated nonlinear, positive associations between all four TyG‐related metabolic indicators and the risk of CKD. Specifically, the odds ratio (OR) for CKD increased significantly when FBG‐HDL exceeded 5.0 (Figure 4A; OR = 3.0), when TCBI surpassed 3000 (Figure 4B; OR ≈ 2.5), and when TyG‐HDL rose above 0.2 (Figure 4C; OR > 5.0)—the strongest association observed among the four indicators. In contrast, CTI exhibited no statistically significant association with CKD risk below 8.5 (Figure 4D), but displayed a clear threshold effect above this value, with OR = 2.0. Collectively, these findings indicate that elevated levels of TyG‐derived metabolic indices are associated with higher CKD risk, with TyG‐HDL showing the greatest discriminatory capacity and CTI exhibiting a distinct inflection point—suggesting that targeted monitoring and control of these indices, particularly beyond their respective thresholds, may hold promise for CKD risk stratification and prevention.

#### 3.2.4. ROC Analyses

**FIGURE 5:**
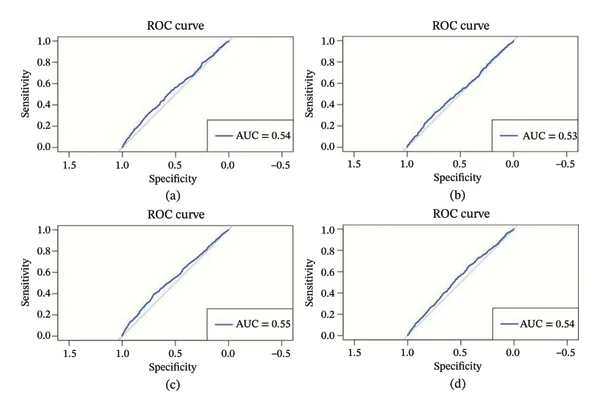
ROC curve.

ROC curve analysis (Figure 5) evaluating the diagnostic performance of four TyG‐related metabolic indicators for CKD yielded modest discriminatory accuracy: FBG‐HDL (AUC = 0.54), TCBI (Figure 5B; AUC = 0.53), TyG‐HDL (Figure 5C; AUC = 0.55), and CTI (Figure 5D; AUC = 0.54). TyG‐HDL achieved the highest AUC among the four indices (0.55), yet all values remained close to the null discriminative capacity (AUC = 0.50), indicating limited utility as standalone screening tools. This finding is consistent with baseline comparisons showing significantly elevated levels of all four indicators in participants with CKD versus those without (*p* < 0.001), but underscores that the magnitude of group‐level difference does not translate into clinically meaningful diagnostic separation. Therefore, rather than relying on any single indicator, a multivariable approach—such as combining these indices or integrating them with established clinical risk factors (e.g., age, eGFR, and albuminuria)—is warranted to improve risk prediction and support early CKD identification.

#### 3.2.5. Subgroup Analyses

**FIGURE 6:**
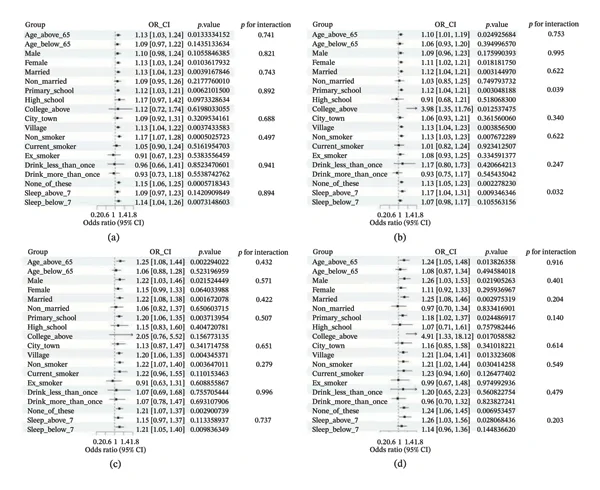
Stratification analysis.

Subgroup analyses (Figure 6) demonstrated consistent positive associations between all four TyG‐related metabolic indicators (FBG‐HDL, TCBI, TyG‐HDL, and CTI) and CKD risk across prespecified demographic and behavioral strata. Specifically, statistically significant associations were observed in participants aged ≥ 65 years, females, married individuals, those with primary education or lower, rural residents, never‐smokers, and those reporting sleep duration > 7 h per day (OR = 1.10–1.26; all *p* < 0.05). Strikingly, effect estimates were markedly amplified in the high‐education subgroup (college or above): TCBI and CTI exhibited substantially elevated odds ratios—3.98 (95% CI: 1.35–11.76) and 4.91 (95% CI: 1.33–18.12), respectively—suggesting potential effect modification by educational attainment. Formal interaction testing confirmed statistically significant effect modification only for FBG‐HDL by education level (primary vs. college or above; *p* = 0.039) and for TCBI by sleep duration (≤ 7 vs. > 7 h; *p* = 0.032); no other stratification variables—including sex, age group, marital status, residence, or smoking status—significantly modified the associations (all *p* for interaction > 0.05). Collectively, these findings indicate robustness of the observed CKD‐metabolic index associations across diverse population subgroups, with education and sleep duration emerging as key effect modifiers warranting further mechanistic and clinical investigation.

## 4. Discussion

This study integrated evidence from both the GBD 2023 database and the nationally representative CHARLS cohort to comprehensively examine the metabolic determinants and population burden of CKD among middle‐aged and older adults in China [22]. Several key findings emerged.

First, at the population level, CKD attributable to elevated FBG has shown a persistently rising burden over the past three decades, and projections indicate an additional upward trend through 2040 [23]. This study found that the age‐standardized burden of CKD attributable to high FBG in China showed a declining trend between 1990 and 2023, yet absolute numbers of deaths and DALYs continued to rise, primarily driven by population growth and aging. The trend was nonlinear: a rapid decline occurred before 2008, followed by a plateau period from 2008 to 2015. In recent years, a slight rebound has occurred, suggesting a recent stagnation in prevention and treatment efforts. This pattern indicates that while early public health interventions and clinical advances may have initially reduced risks, the recent increase in burden likely stems from rising obesity rates, lifestyle changes, or limitations in current prevention strategies. Significant gender disparities exist, with age‐standardized rates consistently higher among men than women, though the rate of decline was greater for men. Decomposition analysis further reveals that while demographic factors are the primary driver of absolute burden increase, epidemiological improvements—particularly among males—significantly offset this growth. This suggests risk reduction strategies targeting males may be more effective, though underlying reasons warrant further investigation. Nevertheless, the contribution of risk improvement remains insufficient to fully counteract demographic pressures. Bayesian age‐period‐cohort models predict that while age‐standardized rates will continue declining, absolute numbers of CKD‐related deaths and DALYs will still rise significantly by 2040 due to population aging. This indicates that without enhanced interventions, the burden of CKD caused by high FBG will continue increasing, placing growing pressure on China’s healthcare system. Future efforts must prioritize early intervention for elevated fasting glucose and enhanced CKD management to address the growing absolute disease burden.

Second, in the CHARLS cohort, only four of the 15 blood glucose‐related composite metabolic indices—TyG‐HDL, TCBI, FBG‐HDL, and CTI—were independently associated with CKD after full adjustment for demographic, lifestyle, and clinical covariates. In contrast, TyG alone and TyG‐related anthropometric indices such as TyG‐BMI, TyG‐WC, TyG‐WHtR, TyG‐BRI, or TyG‐RFM demonstrated no significant associations in fully adjusted models [24]. Third, RCS analyses revealed nonlinear, threshold‐dependent associations for the four significant indices. Finally, subgroup analyses showed overall robustness across demographic and behavioral strata, with effect modification by education level and sleep duration. Together, these findings highlight the importance of metabolic–lipid interactions, rather than simple anthropometric obesity, in the development of CKD among older Chinese adults [25].

These results have several important pathophysiological implications. CKD is increasingly conceptualized as a systemic metabolic disorder rather than a purely renal condition [26]. IR, inflammation, and dyslipidemia are central mechanisms linking metabolic dysfunction to progressive kidney injury. While the TyG index is widely recognized as a reliable surrogate marker of IR, its predictive capacity may vary depending on the biological pathways most relevant to the disease outcome. The present findings suggest that CKD is more tightly linked to combined disturbances in glucose and lipid metabolism—particularly those involving triglycerides (TG) and HDL cholesterol—than to simple IR captured by glucose–TG interactions alone [27].

This helps explain why TyG‐HDL and FBG‐HDL demonstrated strong, independent associations with CKD. HDL plays a crucial role in reverse cholesterol transport, antioxidative activity, and renal endothelial protection. Low HDL levels are known to exacerbate systemic inflammation and oxidative stress, which in turn promote microvascular injury and glomerulosclerosis [28]. When integrated with glucose or triglyceride levels, HDL‐based indices may therefore provide a more holistic reflection of cardiometabolic stress and its impact on renal microcirculation [33]. Similarly, TCBI—a composite indicator incorporating triglycerides, total cholesterol, and body weight—captures combined lipid burden and metabolic load, which may be more directly involved in CKD pathogenesis than BMI or WC alone [34]. CTI, incorporating C‐reactive protein, triglycerides, and glucose, further underscores the role of low‐grade inflammation as a key driver of metabolic–renal interactions.

The lack of significant associations for TyG alone or TyG‐related anthropometric indices requires careful interpretation. In older adults, conventional anthropometric measures such as BMI and WC often fail to accurately reflect metabolic status. Phenomena such as sarcopenic obesity, reduced muscle mass, fat redistribution, and age‐related changes in body composition contribute to a dissociation between body size and metabolic health. Thus, two individuals with similar BMI values may have vastly different metabolic risk profiles [35]. Indicators such as TyG‐BMI or TyG‐WC assume that adiposity amplifies the metabolic impact of IR, which may hold true in younger populations but becomes less valid in elderly cohorts [36]. Moreover, multicollinearity among anthropometric measurements may attenuate their effects in adjusted models, especially when metabolic variables are simultaneously included. These findings reinforce the need to prioritize biochemical markers directly linked to metabolic dysfunction (e.g., TG, HDL, CRP, and glucose) rather than relying on body‐size parameters when assessing CKD risk in aging populations [34, 37].

Beyond their pathophysiological relevance, our findings carry direct and actionable implications for obesity management, nutritional intervention, and innovative CKD prevention strategies. Although conventional anthropometric measures such as BMI and WC did not retain independent significance in fully adjusted models, we contend that this does not diminish the role of adiposity—rather, it underscores that TyG‐derived composite indices more effectively capture the metabolic quality of adipose tissue. These biomarkers integrate visceral adiposity‐driven lipotoxicity, chronic low‐grade inflammation, and IR, all of which are central pathways linking obesity to kidney injury. In elderly populations, where sarcopenic obesity and age‐related fat redistribution frequently dissociate body size from metabolic health, these indices offer a functionally superior alternative to crude anthropometry.

Crucially, each component of these four significant indices—triglycerides, glucose, HDL‐cholesterol, and C‐reactive protein—is directly modifiable through dietary and lifestyle changes. Emerging evidence from randomized controlled trials demonstrates that adherence to Mediterranean‐style diets, rich in unsaturated fatty acids, polyphenols, and dietary fiber, significantly reduces triglyceride and glucose levels while elevating HDL‐cholesterol, thereby improving TyG‐HDL and TCBI scores. Similarly, structured caloric restriction and supervised weight‐loss programs have been shown to decrease TyG‐related indices and concurrently preserve estimated glomerular filtration rate in overweight and obese individuals. These observations suggest that serial monitoring of these composite indices during nutritional counseling could provide patient‐engaging, real‐time feedback on metabolic risk reduction, thereby reinforcing adherence to dietary modifications and physical activity regimens.

From a translational and public health standpoint, the nonlinear, threshold‐dependent relationships identified by RCS analyses offer clinically actionable cutoffs that could be embedded into community‐based screening algorithms. For instance, TYG‐HDL exceeding 0.2 and CTI surpassing 8.5 emerged as potential “red‐flag” thresholds warranting comprehensive metabolic evaluation and targeted lifestyle intervention. We propose a tiered prevention framework: (1) annual community‐based assessment of FBG, TG, HDL‐C, and CRP in adults aged ≥ 60 years; (2) calculation of TyG‐HDL and CTI to stratify individuals into low‐, moderate‐, and high‐risk categories; and (3) for those exceeding predefined thresholds, referral to structured dietary counseling, weight management programs, and intensified glycemic control—with repeated index measurement at 6‐month intervals to track response. This dynamic, risk‐based approach aligns with the global paradigm shift toward precision prevention and personalized metabolic health management, offering a scalable, cost‐effective strategy to curb the escalating burden of obesity‐related CKD in aging societies. Future implementation science research is warranted to evaluate the feasibility, cost‐effectiveness, and long‐term clinical benefits of this index‐guided intervention model across diverse healthcare settings and population subgroups.

Comparing our findings with previous literature, the observed associations between TyG‐derived metabolic indices and CKD are consistent with emerging evidence linking TyG to reduced eGFR, albuminuria, and increased CKD incidence [38]. However, prior studies have seldom evaluated composite indices beyond the classical TyG calculation, and most focused on younger or middle‐aged populations. This study is one of the first to systematically compare fifteen TyG‐based indices in an elderly cohort, thereby expanding the understanding of how distinct metabolic pathways contribute to CKD in later life [39]. Our results also echo findings from recent cardiovascular studies showing that TyG‐HDL and other glucose–lipid composite indicators outperform TyG alone in predicting adverse outcomes. These parallels support a broader shift toward using multidimensional metabolic biomarkers for chronic disease risk stratification [40]. Our research results provide strong support for some previous similar studies. In recent years, some scholars have used meta‐analysis to incorporate a large number of cohort studies and have confirmed that a higher TyG index is independently and significantly associated with an increased risk of CKD [41]. Similarly, studies have also confirmed that an increase in the TyG index can predict the accelerated decline of renal function and the occurrence of CKD [42]. Especially in patients with type 2 diabetes, a high TyG index is closely associated with the rapid progression of diabetic nephropathy, highlighting the importance of early assessment [43]. Furthermore, a recent meta‐analysis has confirmed that an increase in HDL‐C significantly increases the risk of CKD [44]. All of these provide strong evidence for the scientific nature of our research.

From the perspective of public health, the strong associations observed for TyG‐HDL, TCBI, FBG‐HDL, and CTI have several practical implications. First, they highlight the need to integrate simple, low‐cost biochemical markers into community‐based CKD screening strategies, particularly for populations with limited healthcare accessibility. Given the rapid aging of the Chinese population and the substantial projected rise in FBG‐attributable CKD burden, early identification of metabolically vulnerable individuals is increasingly critical. Interestingly, our research findings suggest that after 2020, the burden of CKD caused by high fasting blood sugar among the elderly in China has increased. This might be related to the metabolic sequelae of COVID‐19 in the elderly, resulting in a gradual decline in kidney function among the elderly in China over the past few years. Second, although the ROC analysis indicated modest discriminatory accuracy for all four indices (AUC ≈ 0.53–0.55), this does not diminish their value as components of multivariable predictive models. Metabolic indices are unlikely to function as standalone diagnostic tools for CKD, but their inclusion alongside age, blood pressure, eGFR, and albuminuria may meaningfully enhance predictive performance. Third, the identification of threshold effects in RCS analyses suggests potential clinical cutoffs that could be incorporated into CKD prevention guidelines, although validation is needed in external cohorts [33, 45, 46].

The subgroup findings also offer noteworthy insights. The amplified associations observed among individuals with higher education may reflect greater health awareness, differential health behaviors, or varying exposure to metabolic risk due to lifestyle patterns associated with socioeconomic status. Meanwhile, the modification of TCBI’s association by sleep duration aligns with growing evidence linking sleep deprivation to metabolic dysregulation, inflammation, and renal injury [47]. Such results emphasize that metabolic risk cannot be fully understood without accounting for social, behavioral, and environmental factors. Future studies should explore the mechanistic pathways through which these modifiers operate and consider integrating psychosocial variables into CKD risk prediction models.

The present study has several strengths. The combined use of GBD and CHARLS datasets offers a comprehensive population‐to‐individual perspective, capturing both national burden trends and individual‐level metabolic risk associations [48]. The evaluation of fifteen TyG‐based indices provides a uniquely systematic investigation not previously reported in CKD research. Methodologically, the use of multiple adjusted models, RCSs, subgroup analyses, and interaction tests enhances the robustness of the findings. Moreover, CHARLS provides high‐quality biomarker and sociodemographic data from a nationally representative sample, increasing generalizability to the broader Chinese elderly population [49].

Several limitations warrant consideration. The CHARLS analysis is limited by its cross‐sectional design for some variables, restricting causal inference [23, 26]. Measurement errors in triglycerides, glucose, HDL, and anthropometric variables may influence the accuracy of derived indices. Residual confounding by unmeasured variables—such as dietary patterns, medication use, or genetic predispositions—cannot be excluded [39]. Additionally, CHARLS primarily includes adults aged ≥ 45 years, so findings may not be applicable to younger populations. Finally, although several metabolic indices showed significant associations, their modest AUC values indicate that more complex models integrating multiple biomarkers may be necessary to improve predictive accuracy [50].

## 5. Conclusion

In conclusion, this study demonstrates that composite metabolic indices integrating lipid, glucose, and inflammatory pathways—particularly TyG‐HDL, TCBI, FBG‐HDL, and CTI—are significantly associated with CKD in older Chinese adults, outperforming the traditional TyG index and TyG‐derived anthropometric measures [51]. Rather than negating the role of adiposity, our findings indicate that these indices more effectively capture the metabolic quality of adipose tissue and visceral fat‐driven IR and inflammation—key drivers of CKD pathogenesis in aging populations where sarcopenic obesity often dissociates body size from metabolic health [52].

Importantly, all components of these indices are modifiable through dietary and lifestyle interventions, including Mediterranean‐style diets, caloric restriction, and weight management, all of which have been shown to improve metabolic profiles and preserve kidney function. Given the projected rise in FBG‐attributable CKD burden through 2040, we advocate for integrating TyG‐HDL and CTI into community‐based screening algorithms, using the threshold effects identified in our analyses to guide risk stratification and timely referral to structured nutritional and weight‐management programs. This index‐guided, scalable prevention model offers a cost‐effective strategy to mitigate the escalating burden of obesity‐related CKD in aging societies, warranting future validation through implementation science and randomized controlled trials.

## Author Contributions

The study was designed by Mengjun Bie, Zhenrui Cao, and Yingjiu Jiang. Data collection and analysis were conducted by Jiajie Leng and Li Long The manuscript was prepared by Li Long, Tingting Chen, and Shiying Ye, with figures contributed by Meng Ye and Can Huang. The manuscript was reviewed by Yutong Chen and Xinhong Tian.

## Funding

This study was funded by the Natural Science Foundation of Chongqing, China (No. CSTB2022NSCQ‐MSX0840).

## Disclosure

All authors have read and approved the final manuscript.

## Ethics Statement

The present study is based on data from the CHARLS. The ethical review of the CHARLS project was approved by the Biomedical Ethics Committee of Peking University (IRB: IRB0000105211015). All participants provided written informed consent at the time of data collection. Analyses were conducted on deidentified, publicly available data; therefore, no additional ethical approval was required.

## Conflicts of Interest

The authors declare no conflicts of interest.

## Supporting Information

Additional supporting information can be found online in the Supporting Information section.

## Supporting information


**Supporting Information 1** Supporting Figure 1: joinpoint regression analysis of the burden of CKD attributable to hyperglycaemia among Chinese men, 1990–2022. This figure illustrates the long‐term trend and key turning points in ASMRs for CKD attributable to hyperglycaemia among Chinese men from 1990 to 2022. Joinpoint analysis indicates a sustained and significant downward trend in male ASMR during this period, declining from 740 per 100,000 population in 1990 to 10 per 100,000 population in 2022. ^∗^Denotes statistically significant slope changes in trends (*p* < 0.05). ASMR: age‐standardized mortality rate; APC: annual percentage change. Supporting Figure 2: joinpoint regression analysis of the burden of CKD attributable to hyperglycaemia among Chinese women, 1990–2022. This figure illustrates the long‐term trend in ASMRs for CKD attributable to hyperglycaemia among Chinese women from 1990 to 2022. Joinpoint analysis indicates a significant and sustained decline in female ASMR, from 540 per 100,000 population in 1990 to 180 per 100,000 population in 2022. Marked inflection points (e.g., 2005 and 2015) indicate statistically significant shifts in this trend across different periods (^∗^ *p* < 0.05). ASMR: age‐standardized mortality rate; APC: annual percentage change. Supporting Figure 3: Bayesian age‐period‐cohort (BAPC) model projections of the burden of hyperglycaemia‐related CKD in Chinese men, 2024–2040. This figure, based on a Bayesian age‐period‐cohort (BAPC) model, illustrates historical trends and future projections of hyperglycaemia‐related CKD cases among Chinese men from 1990 to 2040. Model projections indicate that the disease burden within this population will remain largely stable in the coming years, underscoring the need for sustained monitoring and the maintenance of targeted intervention measures for this cohort. Supporting Figure 4: Bayesian age‐period‐cohort (BAPC) model projections of the burden of hyperglycaemia‐related CKD in Chinese women, 2024–2040. This figure, based on a Bayesian age‐period‐cohort (BAPC) model, presents historical trends and future projections for absolute case numbers and age‐standardized incidence rates (per million population) of hyperglycaemia‐related CKD in Chinese women from 1990 to 2040. The model indicates that while absolute case numbers will rise slowly with population growth, age‐standardized incidence rates show a sustained downward trend, projected to remain at a low level by 2040. This outcome suggests that effective epidemiological transitions are partially offsetting the burden increase driven by demographic factors.

**Supporting Information 2** Supporting Table 1: the annual percentage change (AAPC) of CKD attributed to high FBG in elderly Chinese population. Supporting Table 2: analysis of the breakdown of CKD in elderly Chinese individuals attributed to high FBG. Supporting Table 3: the BAPC prediction for the number of elderly Chinese people with CKD attributed to high FBG. Supporting Table 4: the BAPC prediction of the rate of CKD attributed to high FBG in elderly Chinese population. Supporting Table 5: 15 types of TyG indicators and the results of the three models for CKD.

## Data Availability

The data underlying this analysis are from the GBD 2023 study and are publicly available via the Global Health Data Exchange platform. The data used to generate the results of this analysis are available from the corresponding author upon reasonable request.
