# Supplementary material for: TyG‐HDL Outperforms TyG Combined With Obesity Indicators in Predicting Chronic Kidney Disease Associated With High Fasting Blood Glucose Levels in the Chinese Elderly Population: A Two‐Stage Study Based on GBD 2023 and CHARLS Data
Source: Int J Endocrinol. 2026 Sep 7;2026:8245427. doi: 10.1155/ije/8245427 (PMC13549364; doi:10.1155/ije/8245427)
Supplement: Supplementary file 1 [file IJE-2026-8245427-s001.docx]

Supplementary Figure 1: Joinpoint regression analysis of the burden of chronic kidney disease attributable to hyperglycaemia among Chinese men, 1990–2022. This figure illustrates the long-term trend and key turning points in age-standardised mortality rates (ASMR) for chronic kidney disease attributable to hyperglycaemia among Chinese men from 1990 to 2022. Joinpoint analysis indicates a sustained and significant downward trend in male ASMR during this period, declining from 740 per 100,000 population in 1990 to 10 per 100,000 population in 2022. * denotes statistically significant slope changes in trends (P < 0.05). ASMR: age-standardised mortality rate; APC: annual percentage change.

Supplementary Figure 2: Joinpoint regression analysis of the burden of chronic kidney disease attributable to hyperglycaemia among Chinese women, 1990–2022. This figure illustrates the long-term trend in age-standardised mortality rates (ASMR) for chronic kidney disease attributable to hyperglycaemia among Chinese women from 1990 to 2022. Joinpoint analysis indicates a significant and sustained decline in female ASMR, from 540 per 100,000 population in 1990 to 180 per 100,000 population in 2022. Marked inflection points (e.g., 2005, 2015) indicate statistically significant shifts in this trend across different periods (*P < 0.05). ASMR: age-standardised mortality rate; APC: annual percentage change.

Supplementary Figure 3: Bayesian Age-Period-Cohort (BAPC) Model Projections of the Burden of Hyperglycaemia-Related Chronic Kidney Disease in Chinese Men, 2024–2040. This figure, based on a Bayesian Age-Period-Cohort (BAPC) model, illustrates historical trends and future projections of hyperglycaemia-related chronic kidney disease cases among Chinese men from 1990 to 2040. Model projections indicate that the disease burden within this population will remain largely stable in the coming years, underscoring the need for sustained monitoring and the maintenance of targeted intervention measures for this cohort.

Supplementary Figure 4: Bayesian Age-Period-Cohort (BAPC) Model Projections of the Burden of Hyperglycaemia-Related Chronic Kidney Disease in Chinese Women, 2024–2040. This figure, based on a Bayesian Age-Period-Cohort (BAPC) model, presents historical trends and future projections for absolute case numbers and age-standardised incidence rates (per million population) of hyperglycaemia-related chronic kidney disease in Chinese women from 1990 to 2040. The model indicates that while absolute case numbers will rise slowly with population growth, age-standardised incidence rates show a sustained downward trend, projected to remain at a low level by 2040. This outcome suggests that effective epidemiological transitions are partially offsetting the burden increase driven by demographic factors.
